# Efficiency in the operational process: reduction of incorrect entries and guarantee of compliance in the rendering of accounts

**DOI:** 10.31744/einstein_journal/2018GS4200

**Published:** 2018-10-30

**Authors:** Daniela Nobrega Pavão, Monique Buttignol, Adriano José Pereira, Renato Tanjoni, Ederson Haroldo Pereira de Almeida, Patricia Leisnock, Gabriela Sato, Eliézer Silva

**Affiliations:** 1 Hospital Municipal Vila Santa Catarina, São Paulo, SP, Brazil.; 2 Sociedade Beneficente Israelita Brasileira Albert Einstein, São Paulo, SP, Brazil.

**Keywords:** Lean Six Sigma, Triple aim, Process review, Quality of health care, Cost audit, Professional assessment, Lean Six Sigma, Triple aim, Revisão de processos, Qualidade da assistência à saúde, Auditoria de custos, Eficiência profissional

## Abstract

**Objective:**

To verify the impact of the *Lean Six Sigma* methodology in reducing incorrect entries of non-appropriated revenues and expenses.

**Methods:**

Process for the review and application of the *Lean Six Sigma* methodology between December 2015 and September 2016, in a high-complexity general hospital in the city of São Paulo (SP).

**Results:**

A total of 3,756,814 (100%) entries were audited between December 2015 and September 2016. The Sigma level evolved over the course of the process and increased from 3.44 Sigma in December 2015 to 5.92 Sigma in September 2016. Entries classified as non-appropriated revenues and expenses were brought down to 0% at the end of the study.

**Conclusion:**

The use of the *Lean Six Sigma* methodology was efficient in reducing incorrect entries, calculating costs, ensuring compliance in rendering of accounts and accurately determining cost-outcome ratios.

## RESUMO

**Objetivo:**

Verificar o impacto da metodologia Lean Seis Sigma na redução de lançamentos incorretos de receitas e despesas não apropriadas.

**Métodos:**

Processo de revisão e aplicação de metodologia Lean Seis Sigma, no período de dezembro de 2015 e setembro de 2016, em um hospital geral de alta complexidade da cidade de São Paulo (SP).

**Resultados:**

Foram auditados 3.756.814 (100%) lançamentos durante os meses de dezembro de 2015 e setembro de 2016. O nível Sigma foi demonstrado na evolução do processo e aumentou de 3,44 Sigma, em dezembro de 2015, para 5,92 Sigma, em setembro de 2016. Os lançamentos classificados como receitas e despesas não apropriadas chegaram a 0% ao término do estudo.

**Conclusão:**

O uso da metodologia Lean Seis Sigma foi eficiente na redução de lançamentos incorretos, na apuração correta de custos, na garantia de *compliance* na prestação de contas e na acurácia no projeto de custos e desfechos.

## INTRODUCTION

The search for operational efficiency must begin with an understanding of the costing process of health care activities. The auditing of accounts verifies and books all procedures performed with patients, including drugs, materials, and exams, known as entries. Problems in these entries lead to incorrect determination of costs, with potential financial impacts and waste of human resources.

Recently, the Institute for Healthcare Improvement (IHI), an independent, not-for-profit American organization, has proposed the Triple Aim as a set of strategic goals to improve health systems, centered on three dimensions: (1) improve patient care experience; (2) improve population health; and (3) reduce the *per capita* cost of health care.^(^ ^1^ ^)^ In most health systems, these three dimensions are neither integrated nor applied.

The Triple Aim concept also encompasses five components: focus on the patient and the family; redesign of the primary care structure and services; population health management; cost control; and system integration and execution.^(^ ^2^ ^,^ ^3^ ^)^

In order to achieve quality-of-care goals, along with rational expenditure of resources, many organizations, in a supplemental way, use the Lean Six Sigma methodology, which provides quality techniques and principles to achieve performance with virtually no errors. This is because a company’s performance is measured by the Sigma level of its processes, and three or four Sigma levels of performance are accepted as a standard. The tools are applied using a simple model for performance improvement, known as DMAIC, (define-measure-analyze-improve-control - D: define the goals of the improvement activity; M: measure the existing system; A: analyze the system to identify ways to close the gap between the current performance of the system or process and the desired goal, I: improve the system, and C: control the new system).^(^ ^4^ ^)^

The Lean Six Sigma methodology complements the development of health care services and summarizes this approach, leading to an integrated program and improvement of processes. Also, the Lean Six Sigma incorporates diagnostic and analytical tools, and provides good practices and solutions for problems related with waste and unnecessary time consumption.^(^ ^5^ ^)^ The integration of Six Sigma into Lean is a comprehensive, powerful and effective strategy to solve problems and create new processes and products.^(^ ^6^ ^)^

Using resources as rationally as possible and maximizing the ratio between clinical outcomes and operational costs is essential for any health care facility aiming to achieve excellent quality-of-care.^(^ ^7^ ^)^ Thus, understanding the interaction between the different areas is required for a clear understanding of internal processes. These findings provide a basis for decision-making and process performance improvement, with relevant and important impacts on daily operational activities.^(^ ^8^ ^,^ ^9^ ^)^ Little has been described in the literature about how efficiently the operational process can reduce incorrect entries, contributing to specific aspects of costing in health services.^(^ ^10^ ^-^ ^13^ ^)^

## OBJECTIVE

Verify the impact of the Lean Six Sigma methodology in reducing incorrect entries of non-appropriated revenues and expenses.

## METHODS

This study was carried out in a local public hospital in the city of São Paulo (SP), between December 2015 and September 2016. The hospital is part of a partnership between the *Hospital Israelita Albert Einstein* and the City Administration of São Paulo. The hospital began operations in June 2015.

For analysis purposes, only the incorrect entries within said period were included, from all hospital departments without exception, with the view to optimize processes and reduce costs by correcting them.

To understand the operating logic of the hospital investigated, in terms of costs, the different hospital departments were verified and points for improvement were evaluated based on the Lean Six Sigma to optimize the entry process.

To this end, we reviewed the literature on the Lean Six Sigma methodology. Lean Six Sigma was designed to solve problems pertaining to continuous improvement of processes, through the use of several specific tools.^(^ ^4^ ^)^

For analysis purposes, 100% of incorrect entries during the study period were included - not just a sample - from all hospital departments, without exception. Correct entries were not considered in this study.

Initially, the project contract summary was prepared, which was essential to define parameters such as objective, limits, indicator and benefit of the work. The detailing of the process followed the DMAIC steps as demonstrated below.

### D – Definition phase: analysis of the current situation and value stream mapping

The definition phase included an assessment of project needs; team composition; customer demand; how the process was conducted at the time of the study; flow of materials, supplies and information; and waste, as well as how to eliminate it; and consequently, development of a new process flow for the future. Then, we described the problem, project indicator, target, scope and team members.

Here, we used the ARMI Matrix to show the time allocated to this project by each team member, according to their possibilities. The needs were also interpreted based on Critical to Quality (CTQ) indicators, based on the customer’s perspective, and Critical to Process (CTP) indicators, based on the business perspective.

In this step, we built the SIPOC, which is an suppliers, inputs, process, outputs and customers, seeking to understand who the suppliers were, and which were the processes and customers of each department.

The CTQ indicators of the present study were: cost of hospital care procedures; cost *versus* patient outcomes; description of the mechanisms behind the different entries; items without proper appropriation, *i.e* . non-appropriated revenues and expenses (NARE).

The CTP indicators were changes not implemented in a timely manner, before the end of the month; lack of attention by the team when posting entries; constant persistent errors; there are always entries to be evaluated and persistent NARE; previous knowledge of new items to be posted and their parameterization.

**Figure 1:**
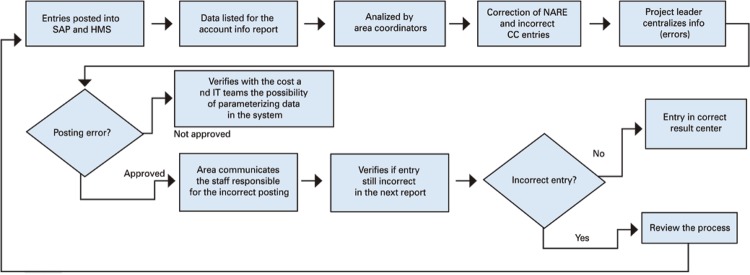
Macroprocess mapping

SAP: system, application and data processing products; HMS: hospital management system; NARE: non-appropriated revenues and expenses; CC: cost center; IT: information technology.

We also carried out macroprocess mapping, *i.e* . a representation of the process as a flowchart for easier understanding. This mapping was done in the Bizagi Process Modeler software and is presented in figure 1 .

### M – Measurement and data collection phase: “What does the process look like today?” - Main causes of the problem

**Figure 2:**
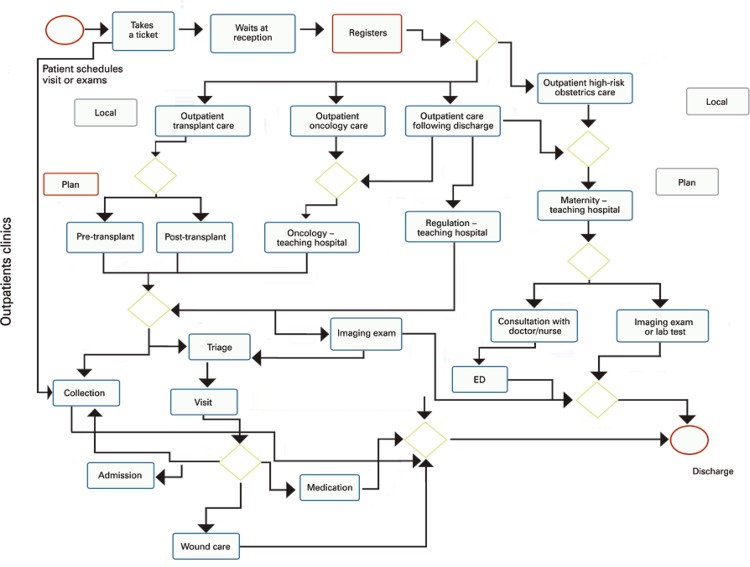
Sample of the flow chart created for each department. In this case, the figure represents the flow chart for the outpatient care service

In this step, we prepared a detailed map of the process. At this point, for the work to continue, we needed all departments to confirm whether the process flow was clear and if metrics were reliable. In addition, we systematically collected data to monitor the measurement and the effects of the action plan implemented ( Figure 2 ).

ED: emergency department.

**Figure 3:**
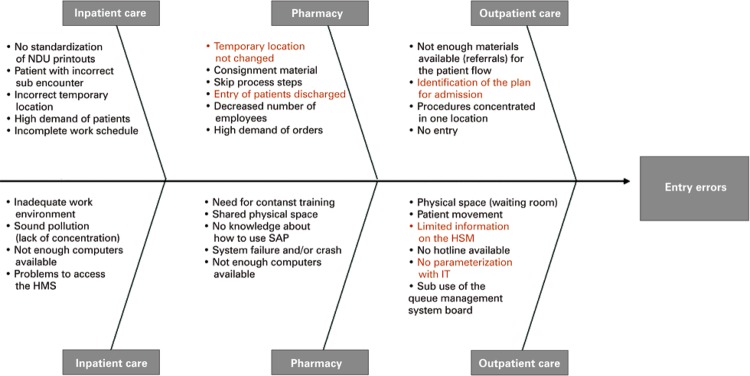
Cause-Effect Diagram for visualization of all causes of a specific problem, considering the main inputs of the process

NDU: urinary output notes; HMS: hospital management system; SAP: system, application and data processing products; IT: information technology; IQMS: Intelligent Queue Management System.

We also applied the Cause-Effect Diagram, aka Ishikawa, for better visualization of all causes of a specific problem based on the main inputs of the process ( Figure 3 ).

Regarding the statistical analysis, 100% of entries were assessed, and the analysis was absolute. Data collection was performed in a planned and validated fashion for all items, to confirm veracity of information.

### A – Analysis phase: tools to observe and analyze the data collected (root cause analysis, waste identification, value-added and non-value added)

**Figure 4:**
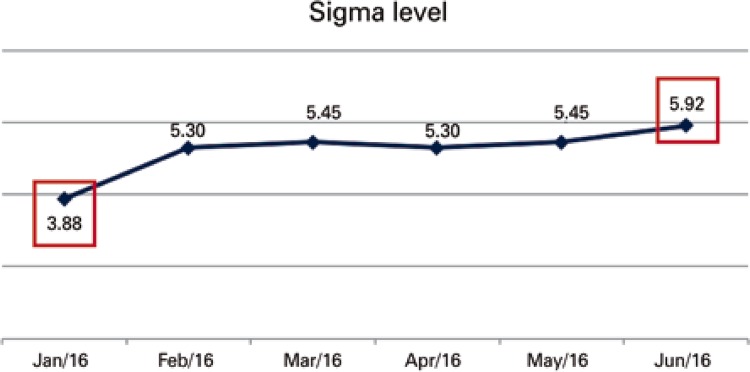
Improvement of the Sigma level month after month. Note the month of January 2016, with a Sigma level of 3.88, and the evolution until June 2016, when the Sigma was 5.92

First, we calculated the initial Sigma level, which demonstrates the proportion of errors. Sigma level 6 means that, for every 1 million occurrences, 3.4 divergences are found ( Figure 4 ).

We had to draw a Pareto Chart to analyze in details the departments with the most divergent entries. After this analysis, the goals were defined using the action plan. The financial gain from the project was calculated considering the number of previous errors and their respective values at the beginning of the project, as well as the proportional number of current entries.

### I – Improvement phase: action plan and confirmation of optimization

In this phase, simple actions, but with great impact, were carried out to achieve the defined objectives, reducing errors in entries. One of these actions was to put a small sign informing the cost center on the computers used for posting entries.

**Figure 5:**
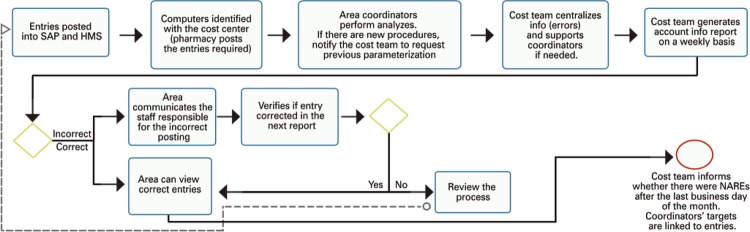
Flow chart with the new Process Map, based on the improvement actions implemented

HMS: hospital management system; SAP; system, application, and data processing products, NARE: non-appropriated revenues and expenses.

After validating all processes, we built a flow chart to show the performance improvement. ( Figure 5 ).

### C - Control phase: statistical control of the process for error-proof methodology

The implemented solutions were controlled on a monthly basis, for data monitoring and auditing of potential discrepancies. This phase included a new calculation of the Sigma level, a control plan, validation of results, standardization of the process and transfer to the process owner.

## RESULTS

According to table 1 , the number of incorrect entries decreased over the course of the analysis, between December 2015 and September 2016. A total of 3,756,814 (100%) entries were audited during this period. The Sigma level was demonstrated and increased from 3.44 Sigma in December 2015, to 5.92 Sigma, in September 2016. The goal of 0% NARE was reached, as initially proposed.

**Table 1:** Statement of non-appropriated revenues and expenses

Month/year	Total number of entries	NARE	NARE (R$)	NARE (%)
December/2015	170,754	1,262	51.171,00	0.74
January/2016	207,490	1,803	71.630,00	0.87
February/2016	298,002	21	16.395,00	0.01
March/2016	401,875	19	4.763,00	0.00
April/2016	510,118	37	2.807,00	0.01
May/2016	332,153	19	4.130,00	0.01
June/2016	399,299	2	2.065,00	0
July/2016	459,897	20	7.354,00	0
August/2016	489,037	7	8.273,00	0
September/2016	488,189	9	2.792,00	0

Table 2 shows a decrease in the loss of R$ 1.8 million when the averages of December 2015 and January 2016 were compared with the last months of the study (July to September 2016). This demonstrates the importance of preventive intervention. This decrease started in February 2016, with significant evolution of NAREs, and a sharp reduction from 1,803 (January 2016) to 21 (February 2016). In subsequent months we can see how the significant decrease in losses continues.

**Table 2:** Calculation of decrease in loss

	Before (December/2015- janeiro/2016)	After (July/2016-September/2016)	Decrease in loss		

			Per entry	Monthly	Annual
NARE/entry (R$)	0.32	0.01	0.312	149,386	1,792,634

NARE: non-appropriated revenues and expenses.

Between February and June 2016, a survey was carried out to identify the problems, and unparameterized entries (33%) and incorrect cost centers (30%) were the most common errors, due to the pharmacy servicing several different units.

## DISCUSSION

The main finding of this study was that the Lean Six Sigma methodology, through its processes, helps decrease incorrect entries found in the auditing of accounts, as well as all inpatient procedures, such as drugs, materials and examinations. The processes involved in the auditing of accounts are directly related with efficiency in reducing incorrect entries, ensuring correct costing, ensuring compliance in the rendering of accounts, and accuracy in costs and outcomes, since they all aim at improving costing efficiency of the operational process. Therefore, there is a need for a tool capable of identifying and generating improvement in health services.

To improve the posting process, the area coordinators were given the necessary explanations, from how to post entries to which are the impacts of errors. The most serious errors were in items that had not been standardized in the system, and these items were adjusted. To this end, a partnership was established with the information technology sector.

Many processes were also carried out improperly. This fact was discovered after mapping of all departments, particularly those with the highest probability for errors, such as outpatient care, gynecological emergency care, medical and surgical clinic, maternal and child care, and pharmacy.

Also, some simple strategies were used to reduce the time wasted, with significant results. For example: some entries were posted under the wrong cost center, such as operating theatre and delivery room, which are considered temporary sites. Therefore, the cost center of each incorrect entry in the system was manually adjusted.

The integration between Lean and Six Sigma is increasingly evident.^(^ ^14^ ^)^ In theory, the idea is to use Lean focusing primarily on eliminating waste and increasing process speed,^(^ ^15^ ^)^ and Six Sigma to reduce variability - and hence defects.^(^ ^4^ ^)^

Lean management is not a new concept, but its implementation in health care services is relatively new.^(^ ^16^ ^)^ Many aspects of the Toyota Production System as well as other tools can be applied to healthcare delivery processes.^(^ ^16^ ^-^ ^19^ ^)^ Some future-oriented health care facilities, such as the Virginia Mason Medical Center (Seattle, W Seattle, Washington, USA), are currently leading the way, showing that this management strategy can reduce waste in healthcare services, with results similar to those of other industries.^(^ ^20^ ^)^ And now our service has been following suit.

Also, the use of this tool showed that the most important factors were related with incorrect entries allocated to transitory NARE cost centers when the same patient visited the hospital several times. It was therefore necessary to effectively and accurately monitor the reasons for these errors. At the time, we anticipated that the number of monthly visits would increase, as new services began operations.

In addition to NAREs, entries were made under the wrong cost center, and we had to ensure the correct appropriation of all divergent entries, to minimize the impact on the cost *versus* patient outcome analysis.

The effective implementation of Lean Six Sigma requires a cultural change that can only happen with top management commitment. In our study, the top leadership played a key role by monitoring, engaging and educating the entire team using strategies, such as weekly meetings with area coordinators to announce results and set targets − this was the most effective measure observed.

Our hospital is inspired by the Triple Aim concept, which includes performance optimization for health systems and the development of new projects to simultaneously pursue three goals: improve patient care experience (including quality and satisfaction); improve population health; and reduce the *per capita* cost of health care.^(^ ^2^ ^)^

When associating Lean Six Sigma and the Triple Aim, one can say that both:

– Improve patient care experience: by reducing rework, less time is wasted, and the staff has more time for the patients. It is possible to improve the patient’s experience based on reports from advisory board meetings (meetings with the leadership, staff, patients and family members), held every two months in each department.

– Improve population health: by optimizing posting processes, costing is more effective, leading to more effective budgeting, improving planning and decreasing variability in the results. This all improves how financial resources are applied for the benefit of the population.

– Reduce the *per capita* cost of health care: the correct appropriation of undue entries contributes to the cost and outcome project of the Institutional Development Program of the Unified Health System (PROADI-SUS - *Programa de Desenvolvimento Institucional do Sistema Único de Saúde* ), an initiative of the Ministry of Health aimed to strengthen the public health system through partnerships with renowned non-profit hospitals.^(^ ^3^ ^,^ ^21^ ^)^

Without a receptive culture, the principles of the Lean Six Sigma would not be possible. IHI believes that many management and operation tools from other industries can be successfully applied to health care. The promise to reduce or eliminate wasted time, money and energy in health care, and the creation of an efficient and effective system that truly meets the needs of patients (the “customers”) are essential and aligned with the principles of the Lean Six Sigma.^(^ ^20^ ^)^

In our study, we measured, reported and compared results, which currently seems to be the best way to create value in health care, by improving outcomes and making the right choices in terms of the cost/outcome ratio for each patient.^(^ ^22^ ^,^ ^23^ ^)^

The main limitation of this study was the integrated system used for posting at the facility investigated. Some of the improvements proposed could not be implemented due to restrictions in the system.

The implementation of all recommendations without increasing the head count or expanding the project team was a challenge. To achieve this, we had to organize the process and set priorities when faced with unexpected setbacks over the course of the process. In the end, there was no additional expenses in any cost center; resources could be reused and reallocated for better results; and a new culture was created in our service. We carried out the transfer to the cost department, which must now monitor and conduct the audits required, to ensure continuity of the improvements implemented. No statistical tests were performed in this study; the analysis of the entries was absolute, *i.e* . all entries were audited, analyzed and corrected, and the number of incorrect entries was brought down to nearly zero. Therefore, there was no statistical analysis to evaluate statistically significant differences, but the results showed a major improvement in the process.

## CONCLUSION

The Lean Six Sigma methodologies were effective in reducing incorrect postings allocated as non-appropriated revenues and expenses, correct costing, compliance assurance, and accuracy in costs versus outcomes. The initially proposed reduction target was reached, which demonstrates the improvement in the posting process. The closer to Six the Sigma level, the better the excellence of the service, which demonstrates that the data can be relied upon to show the relation between costs and patient outcomes at the hospital investigated.
